# Microbial Quality of Leafy Greens Grown Under Soilless Production Systems

**DOI:** 10.3390/pathogens14090943

**Published:** 2025-09-18

**Authors:** Robert Korir Cheruiyot, Abraham Fikru Mechesso

**Affiliations:** Laboratory of Food Safety and Microbiology, Agricultural Research and Development Program (ARDP), Central State University, Wilberforce, OH 45384, USA

**Keywords:** aquaponic, hydroponic, food safety risks, microbial diversity, soilless agriculture

## Abstract

This review examines the microbiological diversity and food safety implications of soilless production systems, particularly aquaponics and hydroponics, which are gaining popularity as efficient methods for producing fresh produce in controlled environments. Despite their advantages, a limited understanding of the microbiological quality and potential food safety risks associated with leafy greens grown in these systems remains. By analyzing published studies, we summarize evidence of microbial contamination in aquaponic and hydroponic environments and their crops, noting that various factors may facilitate pathogen survival and spread to edible plant parts. The operational practices and environmental conditions can promote pathogen introduction through multiple routes, including contaminated fingerlings, fish feed, recirculating contaminated water, pest intrusion, improper handling, and poor worker hygiene. The studies reviewed detected pathogens that pose public health risks, including *Salmonella* spp., *Listeria monocytogenes*, *Pseudomonas aeruginosa*, and Shiga toxin-producing *Escherichia coli* O157:H7, as well as various molds. These potentially contaminated fresh produces are often consumed raw, presenting significant food safety and public health risks that demand further investigation and mitigation strategies to ensure consumer protection while maintaining the benefits of soilless agriculture.

## 1. Introduction

Soilless farming is a cultivation method in which plants grow without soil, receiving essential nutrients directly through the water supplied to their roots [1,2]. It is considered a viable alternative and supplemental method to produce fast-growing crops, particularly leafy greens, herbs, and cannabis. Advances in the development and diversification of growth media with desirable physicochemical properties have significantly contributed to the increased interest in soilless farming systems [3]. Notably, soilless cultivation substantially reduces agricultural water usage [4], making it particularly suitable for regions with limited arable land or infertile soils [5]. Due to ongoing advancements in science and technology, as well as growing investment interest, soilless crop production is expected to expand considerably in the coming decades [6,7].

Soilless farming systems offer several advantages over conventional agriculture, including higher productivity, enhanced environmental sustainability, and improved food safety and security. These systems are recognized for their economic efficiency, optimal nutrient utilization, high planting density, and superior yield and product quality [5,8]. For instance, Bulgari et al. (2016) [9] reported higher mineral content in lettuce and sweet basil grown hydroponically compared to those cultivated in conventional soil-based systems. Soilless farming facilitates local food production, particularly in urban areas, as it is not constrained by climate or geography [10]. It also minimizes microbial contamination risks [11] and avoids challenges associated with soil, such as soil-borne pathogens and reduced productivity because of low soil fertility [3,12]. Furthermore, soilless systems eliminate the need for pesticides, herbicides [12,13], and animal manure. Indoor soilless cultivation provides a controlled environment that restricts access by pests, domestic animals, and wildlife, thereby reducing microbial contamination risks [14,15,16].

Hydroponics, one of the most widely used soilless cultivation methods, involves growing plants in soil-free substrates or directly in nutrient solutions [12,17]. Based on the techniques for distributing nutrient solutions to the plant roots, hydroponic systems are categorized into open or closed systems. In open systems, excess nutrient solutions are drained as waste, whereas in closed systems solutions are recovered, replenished, and recycled, improving water and nutrient efficiency [18,19]. Closed systems may promote the accumulation of organic matter (from water, nutrients, and root exudates) and biofilm formation [20], creating favorable conditions for pathogenic microorganism persistence and spread [21,22]. Biofilms are particularly resistant to standard cleaning and disinfection, allowing pathogens to persist even after water replacement [23,24].

Aquaponic, on the other hand, integrate fish farming and crop production within a recirculating aquaculture system, and it is considered a sustainable agricultural approach [6]. The system involves a complex interaction involving fish, bacteria, plants, and water conditions [25]. Aquaponic outperform hydroponic in water and nutrient efficiency, making it ideal for areas with limited water supply [26,27]. The nutrient-rich water from the fish tank contains elevated levels of toxic ammonia. This ammonia is converted into non-toxic nitrate, a readily available nitrogen source for plants, via nitrification by *Nitrosomonas* and *Nitrobacter*. Subsequently, this treated water is circulated to the aquaponic chamber where crops are grown. While fish waste provides essential nutrients (e.g., nitrogen, phosphorus) and reduces reliance on chemical fertilizers, supplementation of potassium, calcium, and iron is often necessary [27,28]. Commonly cultivated fish species in these systems include Tilapia (*Oreochromis niloticus*), Catfish (*Ictalurus punctatus*), Trout (*Oncorhynchus mykiss*), and Carp (*Cyprinus carpio*) [27].

Although soilless systems contribute to solving food security and safety challenges, they are not without drawbacks. Energy consumption remains a primary concern. Soilless systems located in cold climates could significantly contribute to environmental degradation [29]. In addition, pathogenic microorganisms, which can potentially be introduced into the system, present food safety risks [30]. Crops in soilless systems may become contaminated through multiple pathways, including external root colonization, internalization, and subsequent translocation to edible plant tissues. Additional contamination routes include exposure to contaminated water, poor worker hygiene, or transmission via vectors such as pests and wild animals [16]. 

Leafy greens from aquaponic and hydroponic systems have been associated with foodborne pathogens and outbreaks globally [31,32]. Human pathogenic bacteria such as *Escherichia coli* O157:H7, *Salmonella* Typhimurium, and *Listeria monocytogenes* have been reported to contaminate lettuce and sprouts in these systems, leading to recalls [33,34,35]. From 2010 to 2020, the U.S. reported 245 produce-related foodborne pathogen outbreaks and illness cases, affecting 7,140 people and causing 21 deaths [32]. Of these, 66 outbreaks were associated with *Salmonella*, *Listeria*, and Shiga toxin-producing *E. coli* in leafy greens that led to 15 fatalities [32]. These findings suggest that operational practices in these systems may create opportunities for pathogen contamination and dissemination [36]. 

Despite the increasing adoption of soilless farming, research on its associated food safety risks remains limited. Unlike conventional farming, where pathogenic microorganisms and their contamination pathways are well-characterized, the microbial hazards associated with soilless systems remain poorly understood. This review provides an evidence-based assessment of public health and food safety risks linked to fresh produce from soilless production environments, focusing on aquaponics and hydroponics. In view of the aforementioned facts, the goal of the present review is to explain the public health risks associated particularly with leafy greens grown in aquaponic and hydroponic systems. The specific objectives are to identify the microbes that have been found in leafy greens produced in aquaponic and hydroponic systems, especially those that present food safety and public health risks, and to identify the main factors or sources associated with the introduction of those pathogenic microbes in these systems. The findings will contribute to a better understanding of microbial risks in aquaponic and hydroponic systems and thus support the development of targeted safety guidelines for these emerging practices.

## 2. Methods

### 2.1. Literature Search

**Figure 1:**
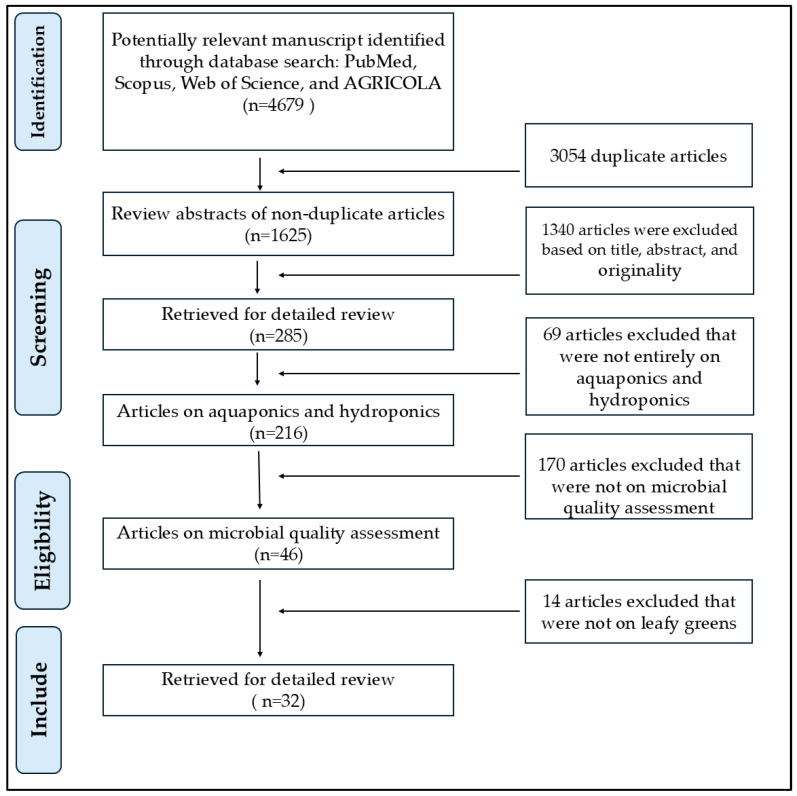
Schematic diagram showing the selection of research studies throughout the process of the systematic review.

This literature review was focused on peer-reviewed studies published between 1999 and 2025. We conducted a narrative review with a systematic search strategy of manuscripts on soilless agricultural production systems across multiple scientific databases, includingPubMed ((https://pubmed.ncbi.nlm.nih.gov, accessed on 12 April 2025), Scopus (https://www.scopus.com/home.uri?zone=header&origin=sbrowse, accessed on 12 April 2025), Web of Science (https://webofscience.zendesk.com/hc/en-us, accessed on 12 April 2025), and AGRICOLA (https://www.nal.usda.gov/agricola, accessed on 12 April 2025) (Figure 1). The search employed MeSH terms and keywords such as “microbial communities,” “aquaponic,” “soilless,” “hydroponic,” “leafy greens,” “foodborne pathogens,” and “microbial food safety.”

To refine the search, we excluded studies containing the terms “field” or “conventional” and focused on controlled-environment crop production. Manuscripts were screened based on titles and abstracts, followed by backward and forward citation tracking of key manuscripts to identify additional relevant literature. Studies were included only if they examined microbial contamination in aquaponic or hydroponic systems, with particular emphasis on leafy greens and fresh fruits. This approach ensured a comprehensive evaluation of available literature while summarizing key findings on pathogens associated with fresh produce in aquaponic and hydroponic production systems. In total, we reviewed 32 peer-reviewed research articles on the microbial profiles of leafy greens grown in aquaponic or hydroponic environments (Figure 1). 

### 2.2. Limitations

The keywords used for searching databases were kept broad to maximize the scope of retrieved articles, and multiple methods of finding sources were used to avoid the limitations of each approach. Even with precautions in place, this review has biases inherent to the search process. The limitation of sources to those published in English excludes any non-English sources. In addition, the keywords chosen for the database searches could have introduced limitations in the search results. However, a concerted effort was made to reduce these shortcomings, and the results are believed to accurately represent the available literature on the subject matter.

## 3. Results and Discussion

### 3.1. Soilless Systems and Associated Microbes

Microbial communities and diversity may vary across different compartments (fish tanks, biofilter, and plant beds) of soilless production systems [37]. Due to the interconnected nature of these components, localized microbial populations interact and influence each other directly or indirectly through metabolic byproducts [38,39]. Schmautz et al. (2022) [38] observed distinct microbial composition in fish feces compared to those in other system components of aquaponic systems, including plant root zones, despite being part of the same aquaponic system. In contrast, other studies report that bacterial diversity remains relatively stable across aquaponic compartments, with community structure primarily influenced by biotic factors (e.g., plant species, fish type, stocking density) and abiotic factors (e.g., pH, alkalinity, temperature, and nutrient concentrations) [40]. Interestingly, various studies have shown that microbial communities in the root zone of aquaponic and hydroponic compartments often resemble those isolated from traditional soil–plant ecosystems [37,38,41], suggesting potential overlaps between soilless and soil-based microbiomes.

#### 3.1.1. Beneficial Microbes

Beneficial bacteria are essential components of the soilless agricultural systems [42]. In hydroponic systems, *Bacillus* spp., *Gliocladium* spp., *Trichoderma* spp., and *Pseudomonas* spp. are widely recognized for their biocontrol properties [43]. In addition, *Bacillus* spp., *Pseudomonas* spp., and *Streptomyces griseoviridis* have been suggested to reduce or prevent the effect of phytopathogens [44] or enhance plant growth [45,46]. In aquaponic systems, nitrifying bacteria play a crucial role in breaking down leftover feed and solid fish waste, converting them into nutrients that plants can readily absorb and utilize [37]. Nitrosomonas convert ammonia emanated from the fish waste into nitrite, whereas nitrobacteria oxidize the nitrite into nitrate, which is a readily available growth nutrient for plants [47]. This microbial nitrification process not only facilitates nutrient assimilation but also ensures water purification through nutrient uptake, enabling efficient recirculation in closed-loop aquaponic. Recirculation of water in soilless systems raises food safety concerns because it can serve as a potential contamination source and an easy mode of pathogen spread to the edible parts of produce [48]. Overall, these observations underscore the indispensable role of beneficial bacteria in enhancing the productivity and sustainability of soilless agricultural systems, particularly hydroponics.

#### 3.1.2. Pathogenic Microbes 

Pathogenic microorganisms of human and plant origin have been reported to colonize, survive, and persist in soilless environment and threaten food safety and public health [49]. Pathogen introduction can occur via contaminated plant materials, growth media reuse, vectors like insects and rodents, as well as poor hygiene practices [50]. The complex interactions among fish (in aquaponic systems), plants, and humans create microbial growth conditions that significantly constrain pathogen control options in soilless systems, particularly in aquaponics [51,52,53]. 

Notable foodborne pathogens, including *Salmonella* spp., *L. monocytogenes*, and *E. coli* O157:H7, have been detected in leafy greens cultivated in hydroponic and aquaponic systems [53,54,55]. A study by Mohammad and colleagues (2022) [56] on microbial surveillance of hydroponically grown lettuce identified *Salmonella* spp., *E. coli* O157:H7, *L. monocytogenes*, and *Staphylococcus aureus*, which are frequently associated with foodborne outbreaks (Table 1). Similarly, Wang et al. (2020) [57] reported Shiga toxin-producing *E. coli* contamination on the leaf surfaces of lettuce, basil, and tomatoes that were grown in aquaponic and hydroponic systems. *Pseudomonas aeruginosa*, which is commonly associated with pneumonia, bloodstream infection, and wound infection in humans, has been also detected in kale and broccoli microgreens, and lettuce grown in an aquaponic farm in the US [58]. The detection of these pathogens challenges the prevailing assumption that controlled-environment agriculture automatically yields safer produce. 

Multiple studies have also documented a wide array of potentially harmful microorganisms in soilless production systems, challenging the assumption that these controlled environments inherently produce safer crops (Table 1). Research has consistently identified microbial contaminants, including total coliforms, generic *E. coli*, various yeast and mold species in hydroponically grown lettuce [59,60,61], with *Enterobacteriaceae* family members detected throughout plant tissues [62]. More alarmingly, many studies have also isolated recognized foodborne pathogens, including *Aeromonas* and *Shigella* species, from fresh produce grown in soilless production systems [58,63] (Table 1). Furthermore, the persistence of both mesophilic and psychrophilic bacteria across different soilless cultivation platforms [37] demonstrates the adaptability of microbial communities to various environmental conditions within these agricultural systems. These collective findings underscore the ongoing food safety challenges associated with soilless farming methods and emphasize the critical need for continued research into effective pathogen mitigation strategies that address the unique microbial ecology of these production environments.

**Table 1:** Summary of literature reviewed on microbes detected in leafy greens produced under aquaponic and hydroponic systems from 2019 to 2025.

Microbes	Source System/Crop	References
Mesophilic aerobic bacteria, enterobacteria, and psychrophilic bacteria	Aquaponic (rainbow trout: *Oncorhynchus mykiss*) and hydroponic: Lettuce	Edgar Wilber et al., 2019 [64]
Coliform, Yeast, and Filamentous fungi	Hydroponic and Aquaponic	Artimová et al., 2023 [36]
Coliforms, Coliforms, *E. coli*, *Listeria*, *Salmonella*	Hydroponic: Bell peppers	Avila-Vega et al., 2014 [65]
*Pythium aphanidermatum* and *P. dissotocum*	Greenhouse: Spinach [66]	Bates, 1984 [66]
Total coliforms and thermotolerant bacteria	Aquaponic: Lettuce	Bianchini et al., 2020 [54]
Aerobic bacteria, coliform bacteria, and yeast	Hydroponic: Lettuce	Dankwa et al., 2020 [67]
Coliforms, yeast, and mold	Aquaponic: Lettuce	Dankwa et al., 2021 [55]
*Pseudomonas* spp. and *Clostridium* spp.	Hydroponic: Microgreens	Dong and Feng, 2022 [58]
*P. aeruginosa* and *Aeromonas hydrophila*	Hydroponic- Lettuce	Dong and Feng, 2022 [58]
*P. aeruginosa* and *A. hydrophilia*	Aquaponic (Nile Tilapia)	Dorick et al., 2024 [52]
*Verrucomicrobia*, *Proteobacteria*, *Planctomycetes*, *Nitrospire*, *Gemmatimonadetes*, *Fusobacteria*, *Firmicutes*, *Cyanobacteria*, *Chloroflexi*, *Chlorobi*, *Bacteroidetes*, *Actinobacteria*, and *Acidobacteria*.	Aquaponic	Eck et al., 2019 [37]
*Luteolibacter*, *Flavobacterium*, *Nitrospira*, *gammaproteobacteria*, *Flavobacterium*, *Pseudomonadaceae*, and *Sphingomonadaceae*	Aquaponic (Tilapia): Lettuce.	Eck et al., 2021 [41]
*Actinobacter*, *Pseudomonas, Shigella*, and *Aeromonas* genera.	Aquaponic (Mozambique tilapia: *Oreochromis mossambicus*)- Lettuce-	Kasozi et al., 2022 [63]
*L. monocytogenes*	Hydroponic: Lettuce	Kyere et al., 2019 [68]
*Listeria* spp. and *E. coli*	Hydroponic: Tomato	Lopez-Galvez et al., 2014 [69]
Generic *E. coli*, *coliforms*, *Salmonella* spp., *E. coli* O157:H7, *L. monocytogenes*, *S. aureus*, yeast and mold	Hydroponic: Lettuce	Mohammad et al., 2022 [56]
Coliforms, *Enterobacteriaceae*, anaerobic mesophilic bacteria, lactic acid bacteria, *Pseudomonas* spp., *enterococci*, yeasts and molds	Aquaponic (Tilapia) and hydroponic: Lettuce	Nissen et al., 2021 [62]
Enterobacteriaceae, coliforms, *E. coli*, and *Salmonella*	Hydroponic: Tomato	Orozco et al., 2008 [70]
Mesophilic bacteria, yeasts and molds, and *Enterobacteriaceae*	Hydroponic: Lettuce	Scuderi et al., 2011 [61]
Mesophilic bacteria, coliforms, yeast, and mold	Hydroponic: Lettuce	Selma et al., 2012 [60]
Coliform, *E. coli*, yeast, and mold	Aquaponic: Lettuce	Sirsat and Neal, 2013 [59]
Mesophilic *bacteria*, coliforms, and *Salmonella*	Hydroponic: Lettuce	Tham et al., 2021 [71]
Shiga toxin-producing *E. coli*	Aquaponic (Nile tilapia: *Oreochromis niloticus* L.) and hydroponic- Lettuce, basil, and tomato	Wang et al., 2020 [57]
Coliforms and *E. coli*	Hydroponic: Cucumber	Xu and Warriner, 2005 [72]

### 3.2. Factors Influencing Human Pathogenic Microorganisms in Soilless Systems

The significant knowledge gap regarding key factors affecting human pathogen contamination in soilless systems complicates the microbial contamination prevention or management approaches. In addition, the implementation of physical, chemical, or antibiotic control measures in soilless systems is particularly challenging due to their potential risks to fish, plants, and humans [41].

#### 3.2.1. Environmental Conditions

The unique characteristics of an aquaponic or hydroponic system, including water recirculation, coupled plant-fish environments for aquaponic, and optimal growth temperatures, create favorable conditions for microbial proliferation. In particular, a closed-loop water system can facilitate the continuous spread and growth of microorganisms, posing a particular challenge [41]. Several studies have detected pathogenic bacteria and fungi such as *S.* Typhimurium, *E. coli*, *Aspergillus flavus*, and *Candida albicans* in aquaponic and hydroponic environments (Table 2). These pathogens have been isolated from various sources, including water samples (reclaimed water, surface water, retention ponds) [34,54,57,67,69,72,73], hydroponic conveyor belts [65], and plant growth medium [74]. Notably, Wang et al. (2020) [57] detected highly pathogenic Shiga toxin-producing *E. coli* in the recirculating water of a hydroponic and aquaponic system. Likewise, an FDA (2022) [75] investigation into *S.* Typhimurium outbreaks linked to leafy greens grown in deep-water hydroponic systems identified stormwater retention ponds and nutrient-rich growth media as the most likely contamination sources. In addition, the age of the aquaponic or hydroponic system has been shown to affect the persistence of some bacterial species, such as *E. coli*. However, Dorick et al. (2021) [51] found no significant correlation between system age and the presence of key human pathogens. These observations highlight the importance of monitoring environmental reservoirs such as untreated groundwater, surface water, and partially treated reclaimed wastewater as potential entry points for pathogenic microorganisms into soilless systems. Once introduced, these pathogens can contaminate crops, particularly leafy greens that are usually consumed raw or undercooked, leading to foodborne outbreaks [75,76,77].

#### 3.2.2. Poor Personal Hygiene and Cross-Contamination

Worker handling and management practices significantly influence the microbial composition of fresh produce grown in soilless systems. According to Dong and Feng (2022) [58], cultivation activities, tool usage, personal hygiene, and post-harvest handling practices collectively shape the microbiome profiles of soilless grown produce. Previous studies have identified human pathogens, including *Salmonella*, *E. coli*, and *Pseudomonas*, on the shoes and clothing of personnel working in hydroponic or aquaponic facilities [58,70,73]. The presence of pathogens on workers’ shoes and clothing highlights the role of human activity as a critical point of control. Additionally, wild and domestic animals such as opossums, mice, and goats accessing production facilities have been implicated as potential vectors for pathogen introduction [70,73]. These findings underscore that poor personnel hygiene and animal intrusions can serve as critical sources of human pathogens in hydroponic and aquaponic ecosystems.

**Table 2:** Summary of literature reviewed on sources of pathogenic microorganisms in aquaponic and hydroponic systems between 1999 and 2025.

Source/Sample	Pathogen	System	References
Environment			
Stormwater retention ponds	*S.* Typhimurium	Hydroponic	FDA, 2022 [75]
Puddles	*Salmonella*	Hydroponic	Orozco et al., 2008 [73]
Reclaimed and surface water	*E. coli* and *Salmonella* spp.	Hydroponic	Lopez-Galvez et al., 2014 [69]
Water and fertilizer solutions	*E. coli*	Hydroponic	Lopez-Galvez et al., 2016 [78]
Well water	*E. coli* and coliforms	Hydroponic	Xu et al., 2005 [72]
Substrate (peat moss) and seedling water reservoir	*Coliforms*	Hydroponic	Dankwa et al., 2020 [67]
Conveyor belt	*Salmonella*	Hydroponic	Avila-Vega et al., 2014 [65]
Water	Total and thermotolerant coliforms	Aquaponic	Bianchini et al., 2020 [54]
Water	*E. coli*	Aquaponic	Dorick et al., 2021 [51]
Plant growth medium (hydroton) and water	Yeast, mold, coliform bacteria, and *E. coli*	Aquaponic	Tunçelli et al., 2023 [74]
Recirculating water	Shiga toxin-producing *E. coli*	Hydroponic and Aquaponic	Wang et al., 2020 [57]
Water and biofilm	*Candida albicans*, *C. parapsilosis Aspergillus flavus*, *A. niger*, *Rhizopus*, *Fusarium* sp., *Trichoderma*, and *Penicillium* sp.	Aquaponic	Sheema et al., 2017 [79]
Wild and domestic animals			
Opossums and mice	*S.* Montevideo	Hydroponic	Orozco et al., 2008 [73]
Goat	*Salmonella* serotype F	Hydroponic	Orozco et al., 2008 [73]
Fish: Aquaponic			
Fish: catfish	*Aeromonas* spp., *Pseudomonas* spp. and *Staphylococcus* spp.	Aquaponic	Chitmanat et al., 2015 [80]
Fish feces: Nile tilapia (*Oreochromis niloticus L.*)	Shiga toxin-producing *E. coli*	Aquaponic	Wang et al., 2020 [57]
Poor Personal Hygiene			
Personnel shoes	*Salmonella* serotype F	Hydroponic	Orozco et al., 2008 [73]
Farm worker’s shoes	*E. coli* and *P. aeruginosa*	Hydroponic and aquaponic	Dong and Feng, 2022 [58]
Personnel cloths	*Salmonella*	Hydroponic	Orozco et al., 2008 [73]
Root/seed internalization: Experimental findings			
Inoculated seedlings and uptake from nutrient solution	*E. coli O157:H7*	Hydroponic	Franz et al., 2007 [81]
Uptake from nutrient solution	*E. coli O157:H7*	Hydroponic	Sharma et al., 2009 [82]
Uptake from nutrient solution	*E. coli*	Hydroponic	Warriner et al., 2003 [83]
Seeds soaked in bacterial cell suspension	*E. coli O157:H7 and S.* Typhimurium	Hydroponic	Jablasone et al., 2005 [84]
Plant roots soaked in nutrient solution containing bacteria	*S. Montevideo*	Hydroponic	Guo et al., 2002 [85]

#### 3.2.3. Plant Tissue Damage

Plant tissue damage, whether caused by mechanical injury or phytopathogen infection, can create entry points and favorable conditions for human pathogen colonization [86,87]. Common phytopathogenic fungi, including *Fusarium oxysporum*, *Botrytis cinerea*, and *Gibberella intricans*, induce lesions in leafy greens. These infections not only compromise plant health but also create optimal conditions for secondary colonization by human pathogens [88,89]. Human pathogens such as *E. coli* O157:H7 and *Salmonella* species can thrive on plant surfaces by exploiting readily available nutrients released through damaged tissues. Macarisin et al. (2014) [87] demonstrated the internalization of *E. coli* O157:H7 into spinach through damaged roots in soil-based systems. Similarly, hydroponic studies have documented pathogen uptake in leafy greens: For example, *E. coli* and *Salmonella* uptake and internalization into spinach and lettuce were observed after seeds or plant roots were soaked in bacterial cell suspension [81,82,83,84,85]). A study by Dankwa et al. (2020) [67] further highlighted this risk in hydroponic lettuce, where contaminated seedling substrate plugs transferred coliforms to roots and edible leaves.

#### 3.2.4. Fish Species and Human Pathogens Contamination in Aquaponic Systems

Human pathogens can contaminate, colonize, survive, and persist at high concentrations within fish raised in aquaponic systems [90]. Fish have been specifically linked to contamination by human pathogens such as *Salmonella* and ciguatoxin, a naturally occurring toxin produced by certain algae [91]. *Pseudomonas* spp., *Staphylococcus* spp. [80], and Shiga toxin-producing *E. coli* [57], which are associated with severe infections in humans, were identified from catfish and Nile tilapia raised in an aquaponic system. Additionally, studies have detected fish-associated microbes in aquaponic systems, including *A. hydrophila*, which is linked with gastroenteritis, wound infections, and septicemia, particularly in immunocompromised individuals [52,80].

### 3.3. Microbial Control in Soilless Systems 

Several plant–pathogen control strategies, such as UV radiation, LED treatments, and media filtration, have shown promise in reducing microbial load in plants [92,93] and could potentially be adapted for human pathogen control in soilless systems. Chemical sanitizers such as hydrogen peroxide and sodium hypochlorite are also effective against specific pathogens but come with limitations, particularly in closed systems where such treatments can harm beneficial microbes or alter water chemistry [94,95]. Additionally, the specificity of microbial interactions in these systems means that unintended ecological disruptions may occur, highlighting the need for targeted approaches that minimize collateral effects. Biocontrol strategies and plant immunity enhancers represent another research focus. Biocontrol agents used in plant pathogen control could potentially reduce the incidence of human pathogens if appropriately selected and validated. However, specificity and delivery methods remain key challenges [92,94,95].

## 4. Conclusions

Soilless production systems support sustainable agriculture and help address global food security challenges, especially with the current consumer demand for local and fresh produce. There is also growing interest in urban farming with limited land access, making soilless systems an ideal solution to adopt. While soilless agricultural systems, namely, aquaponics and hydroponics, offer significant advantages for food production, they are not immune to food safety challenges, particularly contamination by human pathogenic microorganisms. Studies have shown that pathogenic microorganisms such as *Salmonella* spp., *Listeria monocytogenes*, and *E. coli* O157:H7 can persist and spread in these systems, posing significant public health risks. These observations underscore the urgent need to develop and implement stringent food safety protocols for policymaking in Good Agricultural/Handling Practices (GAPs/GHPs) and Hazard Analysis Critical Control Points (HACCP), tailored to the unique and complex characteristics of soilless production environments.

Future research efforts should focus on several critical areas to address these food safety concerns. First, comprehensive investigations into pathogen contamination pathways are needed, including identification of primary microbial reservoirs and elucidation of internalization mechanisms in various leafy green cultivars. Second, a detailed understanding of the complex interplay between beneficial microbiota, such as nitrifying bacteria, saprophytes, and pathogenic organisms, is needed. This necessitates a comprehensive examination of how microbial community structure and function are modulated by variations in critical water parameters, including pH, temperature, dissolved gases (O_2_, CO_2_), and reactive nitrogen species. Third, research should prioritize the development and evaluation of biological control strategies, including probiotic applications and bacteriophage therapies, which could suppress pathogens while maintaining system ecological balance. Additionally, the potential of plant-derived antimicrobial compounds, such as essential oils and phenolic compounds, for post-harvest pathogen reduction merits thorough investigation. Finally, the establishment of standardized monitoring protocols and safety thresholds for indicator microorganisms in soilless systems is essential for ensuring consistent food safety outcomes.

## Data Availability

The original contributions presented in this study are included in the article. Further inquiries can be directed to the corresponding author.
